# Will mania survive DSM-5 and ICD-11?

**DOI:** 10.1186/s40345-015-0041-1

**Published:** 2015-12-09

**Authors:** Jules Angst

**Affiliations:** Psychiatric Hospital, University of Zurich, Lenggstrasse 31, P.O. Box 1931, 8032 Zurich, Switzerland

## Abstract

As a natural phenomenon, mania without major depression will of course survive DSM-5 and ICD-11, but following its integration as a diagnosis into bipolar-I disorder (BP-I) in those manuals, BP-I will be more heterogeneous and, paradoxically, will include a unipolar disorder. Furthermore, unipolar mania will no longer be adequately identified and coded as an independent disorder in public health statistics. Mania without major depression, with prevalence rates of 1.7–1.8 %, is even more common than schizophrenia. This brief review of our current, still insufficient, knowledge suggests strongly that pure unipolar mania, as well as mania with minor depressive disorders, should remain important elements of the three-dimensional mood spectrum. Research should focus on dimensional and not on simplified categorical models, which entail a considerable loss of information.

## Current status of mania

*The Diagnostic and Statistical Manual of Mental Disorders DSM-5* does not list unipolar mania (M) as a specific psychiatric disorder but integrates it into bipolar-I disorder by coding manic episodes as elements of BP-I (Angst 2013a). The future *ICD*-*11 Classification of mental and behavioural disorders* will also follow this approach.

Whilst it is well established that, over lifetime, manic episodes are usually associated with major depressive episodes, there are nonetheless some patients, a subgroup, who manifest manic episodes with minor forms of depression (minor or recurrent brief depression or dysthymia) termed Md. There is yet another group who experience manic episodes only, without any depression (M) (Angst 1978).

The removal of mania as a diagnostic category represents a major change in our dimensional model of the mood spectrum. Is it justified?

The dimensional spectrum concept of mood/affective disorders has two complementary dimensions: (1) a proportional mood spectrum, from major depression (D), via bipolar-II (Dm), bipolar-I (MD) to mania with mild depression (Md) and pure mania (M); and (2) a spectrum of severity from psychotic via major and minor syndromes to symptoms (Angst 2007). A third dimension adds temperament and personality disorders (PD), including borderline PD (Angst 2013b). All three dimensions are divided into subgroups for practical purposes, such as treatment decisions, communication, and population registers.

## Modern mania

Debate over the status of mania as a separate disorder is not recent. Although French psychiatrists in the nineteenth century had convincingly identified the forerunner of our bipolar disorder [Falret’s “folie circulaire” (1851) and Baillager’s “folie à double forme” (1854)], other clinicians and scientists continued to regard melancholia and mania as separate conditions. One such sceptic was the German neurologist Carl Wernicke (1848–1905), to whom we owe the modern concept of mania; for Wernicke psychiatric disorders were brain disorders. In 1896, he described mania as *a state of hyperfunction of nerve transmission and activity, as a different disorder from its counterpart, melancholia, defined as hypofunction or afunction, even though the two disorders frequently co*-*occurred* (Wernicke 1906). Based on Wernicke, Karl Kleist (1879–1960) distinguished unipolar from bipolar conditions, but Kleist’s dichotomy fell to Kraepelin’s unitarian concept of manic-depressive psychoses. The separation of unipolar depression from bipolar disorders resulted from the work of Angst (1966), Perris (1966) and Winokur et al. (1969); this classification was refined through the introduction of bipolar-II disorder by Dunner et al. (1976). Mania was distinguished from bipolar disorder in 1978 (Angst 1978).

## Studies on mania

Compared to major depression, unipolar mania is rare, and research on the disorder is consequently rather scanty. Two large epidemiological studies in adolescents and young adults reported prevalence rates of 1.7 % up to age 17/18 (Merikangas et al. 2012) and 1.8 % up to 21–34 years of age (Beesdo-Baum et al. 2009). Most clinical studies dealt with retrospective data and the transcultural findings were sometimes contradictory. Nonetheless, a considerable body of evidence (including epidemiological, clinical, treatment, cardiovascular, and genetic studies) points to the existence of mania as a separate entity (Angst and Grobler 2015). That review, which followed on from the revived interest in the subject, stressed the need for long-term prospective studies and concluded that unipolar mania exists and that subjects with three or more manic episodes without major depressive episodes (MDE) show a good diagnostic stability.

## The evidence

Diagnostic change over time is of particular interest. In patients with MDD it is relatively low. A 25 years’ follow-up of hospitalised patients with MDD found a rate of diagnostic change to BP-I disorder of 1 % per year of follow-up and of 0.5 % per year to BP-II (Angst et al. 2005). For pure mania (*N* = 33) the conversion rate to bipolar disorders was 2.7 % per year, even higher than for MDD. In the Early Developmental Stages of Psychopathology (EDSP) study 3021 adolescents and young adults, aged 14–24 at baseline, were followed up three times until the age of 21–34 years. Half the subjects with initial mania developed MDE and were diagnosed later as having BP-I, but the final prevalence rate of mania was still 1.5 % and of unipolar hypomania 1.8 % (Beesdo-Baum et al. 2009). The cross-sectional US National Comorbidity Survey of 10,123 adolescents (NCS-A) aged 13–18 found 1.7 % M/Md and 2.5 % MD/Dm (Merikangas et al. 2012). Several studies have shown the good diagnostic stability of mania over time (Angst and Grobler 2015). In a very sound 10-year follow-up study of 24 patients with mania, the stability of the diagnosis was 75 % (Xu and Chen 1992). Over a period of 7 years (Yazici et al. 2008), Yazici (2014) found a stability of 88 % in patients with 4+ manic episodes.

Compared to depression, mania has a relatively early onset, with midlife or late-onset mania seemingly the exception. In the prospective epidemiological Zurich Study (probands followed from 20–50 years of age) bipolar-I disorder manifested in all instances before the age of 35. On the other hand, major depressive disorder showed unchanged incidence rates up to age 50, and later incidence of depression is, of course, quite common.

Clinical studies comparing patients with mania with those having bipolar disorder found that mania has an earlier age at onset, higher rates of psychotic symptoms, less rapid cycling, fewer suicide attempts, higher rates of hyperthymic temperament, complete absence of depressive temperament, less anxiety and better long-term functional adjustment (Pacheco Palha and Arrojo 2009; Merikangas et al. 2012). Two studies also found a lower family load for depression (Perugi et al. 2007; Ghaffarinejad et al. 2013).

Evidence also comes from treatment studies: patients with mania responded less well to lithium than those with BP-I but had similar response rates to valproate (Angst and Grobler 2015). Moreover, our study on the association of affective disorders and cardiovascular illness showed that over a 50-year follow-up of 403 hospital admissions for affective disorders the cardiovascular standardised mortality rate (SMR) was highest in patients with M/Md (3.17), followed by BP-I (1.99), BP-II (1.60) and MDD (1.32). The threefold cardiovascular mortality risk incurred by patients with mania may be a consequence of an increased stress burden (lifestyle, excessive activity, short sleep, secondary substance abuse, etc.). Nonetheless, patients with mania in our study lived longer than those with bipolar disorders and depression, because fewer of them died by suicide, shown by the SMRs. In patients with mania, deaths by suicide were confined to the first 10 years of follow-up; in bipolar patients that risk persisted over 25 years, and in patients with MDD it was lifelong throughout the full 50 years of the follow-up (Angst et al. 2013).

There is ample evidence that pure mania is more common in non-Western cultures (Angst and Grobler 2015). It is worth noting that a special code for manic disorder was included in the second edition of the Chinese Classification of Mental Disorders (CCMD-2) following findings of a higher prevalence of mania in that country.

Of considerable interest are the genetic findings from two recent family studies that found a degree of independence of familial transmission of mania and depression. The first study (NIMH) (Merikangas et al. 2014) reported specific familial aggregations of probands’ mania with relatives’ mania (OR = 8.3) and a much lower association of probands’ major depression with relatives’ major depression (OR = 2.5); whereas there was no significant transmission from mania to depression or from depression to mania. In addition, the heritability of mania was higher (0.83) than of any bipolar disorder (0.61) or MDD (0.54); it was lowest for hypomania (0.20) and bipolar-II disorder (0.20). Very similar data were reported from a second study (Lausanne and Geneva) (Vandeleur et al. 2013). The association between probands and relatives was significant for mania to mania (OR = 6.4), for depression to depression (2.0) and for depression to mania (2.0), but non-significant from mania to depression (1.7). Somewhat different conclusions were drawn from the twin study of McGuffin et al. (2003), who reported a substantial genetic correlation between mania and depression (0.65), but found that 71 % of the variance for mania was not shared with depression.

## Conclusions and recommendations

It is abundantly clear that we must continue to be able to describe and distinguish M/Md in the interests of both patients and science. The disappearance of mania as a diagnosis may have serious implications for research in many fields, beginning with epidemiological studies, based on diagnostic patient registers worldwide. It will especially impair brain and genetic research and the investigation of mortality and somatic comorbidity by introducing an unnecessary heterogeneity of diagnostic samples. Research should not be based only on diagnostic groups but also on dimensional descriptive variables, as illustrated by the modern spectrum concept of mood disorders (Angst 2013b), dimensions which include severity, manic and depressive proportionality and temperament (including affective personality disorders).
